# Prevalence of Hypothyroidism in the Population of West Bokaro Coal Mine Area, Jharkhand: A Hospital-Based Observational Study

**DOI:** 10.7759/cureus.28733

**Published:** 2022-09-03

**Authors:** Praveen Kumar, Arnab Mukherji, Ashish Roy

**Affiliations:** 1 Department of Pathology and Laboratory Medicine, Tata Central Hospital (West Bokaro Coal Mines), Ranchi, IND; 2 Department of Medicine, Tata Central Hospital, Ramgarh, IND; 3 Department of Pediatrics, Tata Central Hospital, Ranchi, IND

**Keywords:** opd, west bokaro coal mines jharkhand, hyperthyroidism, tsh, subclinical hypothyroidism

## Abstract

Background

Hypothyroidism is a common endocrine disorder worldwide. Studies on the prevalence of hypothyroidism in different geographical territories of India are sparse. Data on the prevalence of hypothyroidism in India's coal mine areas are lacking. Therefore, we conducted a cross-sectional study to determine the prevalence of hypothyroidism in the adult population living in the coal mine areas of West Bokaro, Jharkhand, India.

Methods

In total, 1484 individuals of both sexes attending the outpatient department (OPD) of Tata Central Hospital, West Bokaro, Jharkhand, with varied symptoms were screened for thyroid-stimulating hormone (TSH) levels from January 2021 to February 2022. The age of the study participants ranged from 15 to 80 years.

Results

In total, 366 participants had hypothyroidism (subclinical as well as overt). The prevalence of hypothyroidism was greater in women than in men. Among the 366 patients with hypothyroidism, 311 were women and 55 were men, and the ratio was 5.5:1. The percentage of the population having hypothyroidism was 24% in this study, which is higher than that reported in other parts of India; however, our results are similar to those of a study conducted in Assam in 2017. Among patients with high TSH levels, 47%, 25%, and 19% had TSH in the range of 5.6-7.5, 7.6-10.6, and 10.6-20 μU/mL, respectively.

Conclusions

Subclinical and overt hypothyroidism are common in eastern India. Patients with undiagnosed fatigue and weight gain must be screened for TSH levels. Hypothyroidism is no longer a rarity, and coal mine areas are no exception to this phenomenon. A population‑based epidemiological study of thyroid disorders in coal mine areas is an urgent need.

## Introduction

Hypothyroidism is a common endocrinological problem worldwide. The global burden of hypothyroidism is significant. In the developed world, hypothyroidism prevalence is approximately 4-5% [1-3] and that of subclinical hypothyroidism is approximately 4-15% [1,3,4]. In India, hypothyroidism is common. Iodine deficiency is the commonest cause of goitre and hypothyroidism in India. Since India adopted the universal salt iodinization programme in 1983, a decline in goitre prevalence has been observed in several parts of the country, which were previously endemic [5-7]. Subclinical hypothyroidism is the most common thyroid disorder in adults and is more common in women and elderly people than in men and young people, and its incidence increases with an increase in iodine intake [8-11]. Due to the asymptomatic nature of subclinical hypothyroidism, the American Thyroid Association has recommended routine thyroid-stimulating hormone (TSH) screening for both sexes at the age of 35 years and every 5 years thereafter. Until now, no guidelines are available in India for TSH screening. A few studies are available on hypothyroidism prevalence in different geographical territories of India.

Hypothyroidism results from low levels of thyroid hormone with varied etiology and manifestations. Untreated hypothyroidism increases morbidity and mortality. The patient's presentation can vary from asymptomatic disease to myxoedema coma. Presently, hypothyroidism can be easily diagnosed with simple blood tests and can be treated with an exogenous thyroid hormone.

This observational study was conducted to determine the hypothyroidism prevalence in patients visiting the outpatient department (OPD) of Tata Central Hospital, West Bokaro, Jharkhand.

## Materials and methods

A single-centre, observational study was conducted in the West Bokaro coal mine area, Jharkhand, India, to study hypothyroidism prevalence among the adult population. Thyroid abnormalities were diagnosed based on laboratory results of TSH. Patients with a history of hypothyroidism and receiving levothyroxine therapy or those with serum TSH > 5.50 μU/mL were considered to have hypothyroidism. The TSH of all the participants was measured at the same laboratory on a chemiluminescent immunoassay platform by using dimension equipment from Siemens. A reference range of 0.3-5.5 μU/mL was considered normal.

The information for the study was collected from patients visiting the OPD at Tata Central Hospital, West Bokaro coal mine area, Jharkhand, from January 2021 to February 2022.

Previous studies have shown that the prevalence of hypothyroidism is 10.9%. This established the proportion under the null hypothesis as 0.11. To calculate the sample size for binomial data, using a non-inferiority alternate hypothesis to infer a relative difference with a type one error of 5% and power of 80%, to detect a minimum detectable difference of 3% and a dropout of 20%, a minimum sample size of 800 will be adequate. The sample size was calculated manually by using one sample proportion formula.

A total of 1484 individuals of both sexes in the age group of 15-90 years were included in the study. These patients presented to the OPD with vague symptoms of weight gain, cold intolerance, menstrual irregularities, lethargy, and fatigue. After a thorough clinical examination, TSH screening was performed on all of them, and the results obtained were analyzed.

All patients in the age group of 15-90 years who consented to participate in the study were included in the study. Patients aged <15 or >90 years were excluded. Furthermore, pregnant women and patients with acute or chronic systemic illnesses were excluded. Moreover, patients taking steroids, lithium, amiodarone, or other drugs that can interfere with thyroid function tests were excluded. Additionally, patients who have undergone thyroid surgery or radiotherapy to the head/neck area were excluded.

A detailed history of any chronic illness and drug intake history (e.g., steroids, lithium, oral contraceptives, or other hormonal therapy) was obtained. A detailed clinical examination was performed to exclude any chronic or acute systemic illness. Laboratory investigations including tests for biochemical parameters (e.g., complete blood count, fasting blood sugar, renal function tests, liver function tests, serum proteins, serum albumin, and electrolytes) were performed where deemed necessary.

## Results

A total of 366 of 1484 (prevalence 24%) participants received a diagnosis of hypothyroidism, which is similar to the findings of a study by Mahanta et al. from Assam Medical College [12] in 2017 (prevalence 24%). Out of 366 participants, 311 were women and 55 were men. Hypothyroidism was found to be more common in women (20.9%) than in men (3.7%) in the overall population (Table 1). Among those with increased TSH levels, TSH was in the range of 5.6-7.5 μU/mL in 47%, 7.6-10.6 μU/mL in 25%, and 10.6-20 μU/mL in 19% (Table 2). The male-to-female ratio of patients with hypothyroidism was 1:5.5. The most common clinical presentation in this study was fatigue (62%), followed by weight gain (19%), body aches (15%) along with constipation, menstrual irregularities, and infertility. Among the 1484 participants, 31 had hyperthyroidism, of which 11 (0.75%) were men and 20 (1.3%) were women. The reason for this may have been overtreatment, which was corrected later with a dose reduction.

**Table 1:** Euthyroid, hypothyroid, and hyperthyroid—gender-based distribution

Level of TSH	Category	Male	Females	Total
0.01–0.3	Hyperthyroid	11	20	31
0.3–5.5	Euthyroid	215	867	1082
5.5 to above	Hypothyroid	55	311	366
Total		284	1200	1484

**Table 2:** Thyroid-stimulating hormone level distribution

Level of TSH (μU/ml)	Male	Females	Total
5.6–7.5	31	142	173
7.6–10.5	9	85	94
10.6–20	14	57	71
21–30	0	7	7
31 to above	1	20	21
Total	55	311	366

Data were collected using MS Excel (Microsoft® Corp., Redmond, WA). Continuous variables were expressed as mean (SD), median, and ranges; categorical variables were expressed as numbers and percentages. The age-wise distribution of the participants is presented in Table 3.

**Table 3:** Age-wise distribution of participants

Age group in years	Male	Females	Total
15–25	30	195	225
26–35	290	37	327
36–45	290	108	398
46–50	47	137	184
51–60	94	187	281
61–70	33	50	83
71–90	12	38	50

## Discussion

Hypothyroidism is a common endocrine disorder in India and other parts of the world. The clinical manifestations of hypothyroidism are varied, including weight gain, cold intolerance, menstrual irregularities, lethargy, and fatigue. The clinical spectrum of hypothyroidism varies from asymptomatic subclinical hypothyroidism through overt hypothyroidism to life-threatening myxoedema coma. Women are more prone to hypothyroidism than men. The prevalence of hypothyroidism was found to be common in the 35-45-year age group (Table 4), which is contrary to that reported in previous studies. In recent studies, overt hypothyroidism has been found to be more common in women than in men.

**Table 4:** Age- and gender-wise distribution of patients with hypothyroidism

Age group in years	Male	Females	Total
15–25	3	46	49
26–35	4	66	70
36–45	3	94	97
46–50	14	30	44
51–60	21	51	72
61 to above	10	24	34
Total	55	311	366

**Figure 1:**
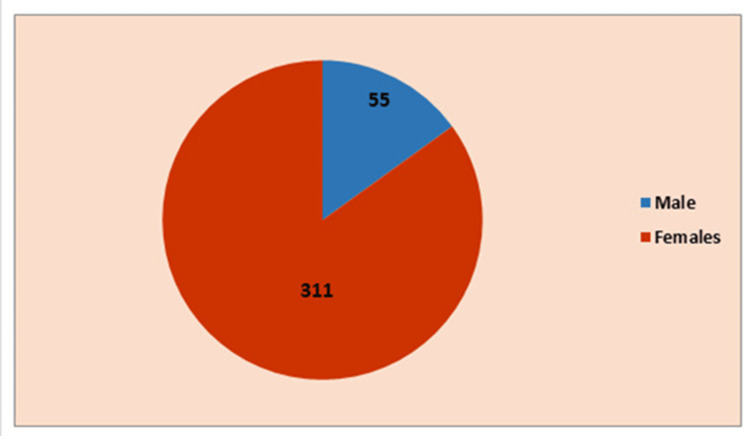
Above 5.5 level of thyroid-stimulating hormone in male and female

The male-to-female ratio in this study was 1:5.5 (Figure 1). Takashi et al. [13] reported a male:female ratio of 1:6 for hypothyroidism, which is similar to that in the present study, whereas John et al. [14] reported the ratio to be 1:4, which is lower than that of the present study. Deshmukh et al. [15] reported a male:female ratio of 1:3.7 for hypothyroidism, which is also lower than that of the present study.

**Figure 2:**
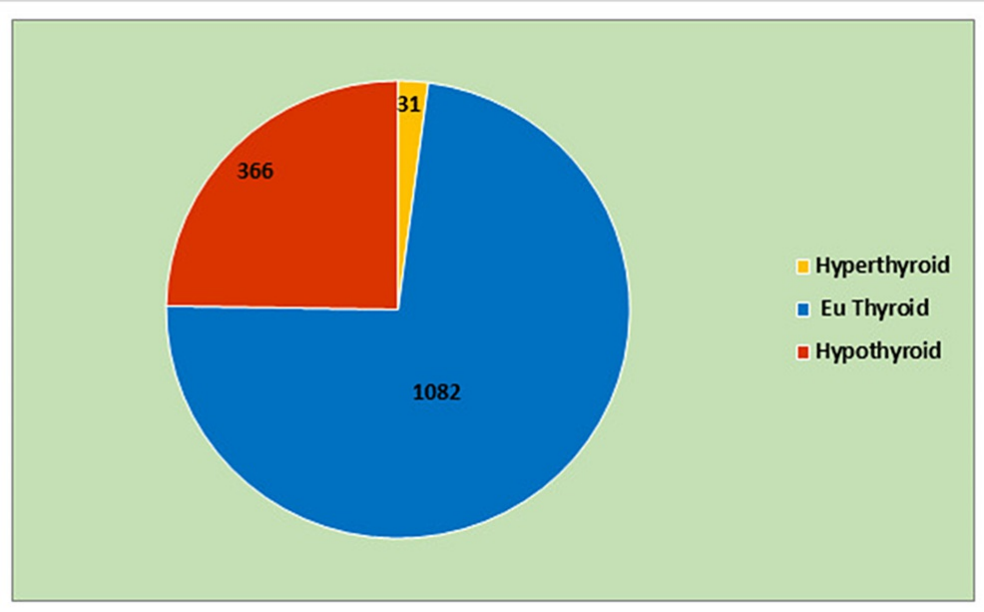
Thyroid status of the study population

The high prevalence of hypothyroidism in the present study is probably due to referral bias because candidates were sent for sampling based on clinical symptoms. Hence, this figure may be higher than the actual prevalence in the general population. However, the high prevalence of 24% (Figure 2) in the current study is similar to the study results of Mahanta et al. from Assam Medical College [12] in 2017.

In an epidemiological study conducted in eight cities in India, Unnikrishnan et al. [16] reported the overall prevalence of hypothyroidism to be 10.95%, which included 7.48% of self-reported hypothyroidism.

Limitations

This study being a cross‑sectional observational study, the study participants were not followed‑up. Moreover, sampling was performed based on clinical signs and symptoms. Furthermore, the study results are subject to referral bias, which may account for the high prevalence of overt hypothyroidism found in our study. Free T3 and free T4 analyses were not conducted in our study, and hence, patients with central hypothyroidism were not excluded. Additionally, the evaluation for autoimmune thyroid diseases with the levels of antithyroid antibodies such as thyroid peroxidase antibodies was not performed.

## Conclusions

Hypothyroidism is a significant health problem in India and worldwide. Our study provides an idea of the prevalence of this entity in the coal mine areas of West Bokaro. Subclinical and overt hypothyroidism is common in eastern India. Hypothyroidism is common in women, usually presenting as vague manifestations. The common presenting complaints are fatigue, body aches, weight gain, constipation, menstrual irregularities, and infertility. Therefore, any individual presenting with undiagnosed fatigue, weight gain, and menstrual irregularities should be subjected to TSH screening. Given the findings of the present study and considering its limitations, a long‑term population‑based, follow‑up study of thyroid disorders in the coal mine areas of India is needed.
